# New Strategy for the Detection and Treatment of *Helicobacter pylori* Infections in Primary Care Guided by a Non-Invasive PCR in Stool: Protocol of the French HepyPrim Study

**DOI:** 10.3390/jcm11051151

**Published:** 2022-02-22

**Authors:** Maxime Pichon, Bernard Freche, Christophe Burucoa

**Affiliations:** 1 Bacteriology Laboratory, Infectious Agents Department, CHU Poitiers, 86021 Poitiers, France; 2 INSERM U1070 Pharmacology of Antimicrobial Agents and Resistances, University of Poitiers, 86073 Poitiers, France; bernard.freche@univ-poitiers.fr; 3 Department of General Medicine, Faculty of Medicine and Pharmacy, University of Poitiers, 86073 Poitiers, France

**Keywords:** *Helicobacter pylori*, primary care physicians, antimicrobials, diagnosis

## Abstract

*Helicobacter pylori* (Hp) infects half of the world population and is responsible for gastric, duodenal ulcers and gastric cancer. The eradication of Hp cures ulcers and prevents ulcer recurrences and gastric cancer. Antibiotic resistance of Hp, and particularly clarithromycin resistance, is the primary cause of treatment failure and is a major concern identified by the WHO as a high priority requiring research into new strategies. Treatments guided by the detection of antibiotic resistance have proven their medical and economical superiority. However, this strategy is severely hampered by the invasive nature of the fibroscopy, since antibiotic resistance detection requires gastric biopsies. The eradication of Hp involves primary care physicians. The objective of this study will be to evaluate the feasibility of a strategy for the management of Hp infection in primary care by a recently developed non-invasive procedure and its non-inferiority in eradication rates compared with the strategy recommended by the French National Authority of Health. The non-invasive procedure is a PCR on stool to detect Hp infection and mutations conferring resistance to clarithromycin allowing a treatment guided by the results of the PCR. We present the protocol of a prospective, multicenter, randomized, controlled interventional study in two arms.

## 1. Introduction

*Helicobacter pylori* (Hp) infection is the most common chronic bacterial infection in the world (50% of the world population, 20 to 30% of the French population) [1,2,3]. Acquired in childhood, most often within the family, this infection persists throughout the individual’s life in the absence of specific treatment [4]. Many infected persons present chronic gastritis, most of the time asymptomatic. Only 5–10% of infected people will develop a gastric or duodenal ulcer, 1–3% gastric cancer (adenocarcinoma or MALT lymphoma) [5]. This represents 80,000 cases of peptic ulcers and 6000 gastric cancers (5-year survival rate of 20%) in France each year [6,7]. Hp is recognized as a class 1 carcinogen by the WHO (800,000 deaths from gastric cancer worldwide each year) [7,8]. Treatment cures peptic ulcers and prevents their recurrence, prevents gastric adenocarcinoma and cures MALT lymphoma without associated translocation. Resistance to the antibiotics used in eradication therapy is the primary cause of treatment failure, followed by poor compliance [9,10].

In Europe *H. pylori* resistance rates in 2018 (on 1211 strains) were 21.4% for clarithromycin, 15.8% for levofloxacin and 38.9% for metronidazole. Prevalence was significantly higher in Central/Western and Southern than in the Northern European countries [11]. In the same year, 20.9% of Hp strains in France were resistant to clarithromycin, 17.6% to levofloxacin, and 58.6% to metronidazole [10,12,13]. The 2017 recommendations of the French National Authority for Health (Haute Autorité de Santé, HAS) placed “guided” and “empirical” treatment modalities at the heart of strategies for Hp eradication [14]. These “guided” treatments following the identification of resistance to certain antibiotics have proven their medico- economic superiority in the eradication of this bacterium [15]. This guided treatment strategy requires gastric biopsies and culture of this difficult-to-culture bacterium and/or a real-time PCR [16,17]. This strategy is severely hampered by the invasive nature of the gastroscopy, the difficulty of the culture and the cost of the PCR. It has been widely demonstrated that treatment guided by resistance detection is more effective, less expensive and better tolerated. The World Health Organization, WHO, has identified Hp resistance to clarithromycin as a high priority requiring research into new strategies. In France, guided treatment represents less than 1% of eradication treatments (Longitudinal Patient Data and French national Health information system, SNIIRAM), despite the recommendations of the HAS and the European and French Societies for Gastroenterology to favor treatment guided by the results of Antibiotic Susceptibility Testing, AST, or PCR.

Serology, a non-invasive test, easy-to-perform and inexpensive, has demonstrated good performances (sensitivity, Sen, over 96% and specificity, Spe, between 60 and 90%). However, it does not allow detection of antibiotic resistance and the active nature of the infection. Similarly, other non-invasive tests have been developed to detect Hp in other, less invasive specimens, such as respiratory specimens (for the urea breath test, used for eradication testing) or stool (stool antigen test) (described or reviewed in refs. [18,19,20,21]). The latter have the same limitations as serology, being limited to the presence/absence of Hp without any precision on the AST profile, crucial for disease management in many countries of the world. Several diagnostic companies have recently marketed PCR kits for the detection of Hp infection and clarithromycin resistance mutations in stool. The performances of the Amplidiag *H. pylori* + ClariR test (Mobidiag, Hologic Inc., Marlborough, MA, USA) for detection of Hp infection and detection of clarithromycin resistance by real-time PCR have been demonstrated [10]. These excellent performances (Sen 96.3% and Spe 98.7%) make it possible to consider using this non-invasive technique for diagnosis and treatment orientation and consequently to be able to carry out guided treatment without recourse to gastro-duodenal endoscopy and biopsies. PCR testing of stool for Hp infection and resistance to clarithromycin is simple and requires few trained personnel. Its non- invasive character may allow it to be used in patients with an indication for the detection and treatment of Hp infection and without an indication for gastro-duodenal endoscopy.

The eradication of Hp strongly involves primary care physicians (general practitioners, GPs). The diagnosis of Hp infections in the general population requires close collaboration between primary care physicians and other specialists (gastroenterologists, pathologists, bacteriologists...). In France, it has been estimated that GPs see almost the entire population of France in their offices at least once a year. Given the prevalence and incidence of Hp, their role is becoming increasingly important [22]. The present project is therefore related to a field problem. The GP’s skills in the prevention of gastric cancer and pathologies of the upper digestive tract are an integral part of the discipline’s professional reference system [23].

The main objective of this study will be to evaluate, in a real-life situation, the feasibility of a strategy for the management of Hp infection by a non-invasive procedure in primary care and its non-inferiority in eradication rate compared with the strategy recommended by the HAS using a combination of serology and endoscopy. The secondary objectives of this study will be: to evaluate the adherence of patients to the care pathway (compliance to endoscopy, treatment, stool collection, serology); to evaluate the follow-up of recommendations in the management of Hp infections by health professionals (GPs and gastroenterologists) (e.g., empirical/guided treatment, evaluation of pre-neoplastic lesions); to validate, in real-life circumstances, real-time PCR in stool in comparison with serology in primary diagnosis and with urea breath test in eradication control.

## 2. Methods

### 2.1. Trial Design and Setting 

**Figure 1:**
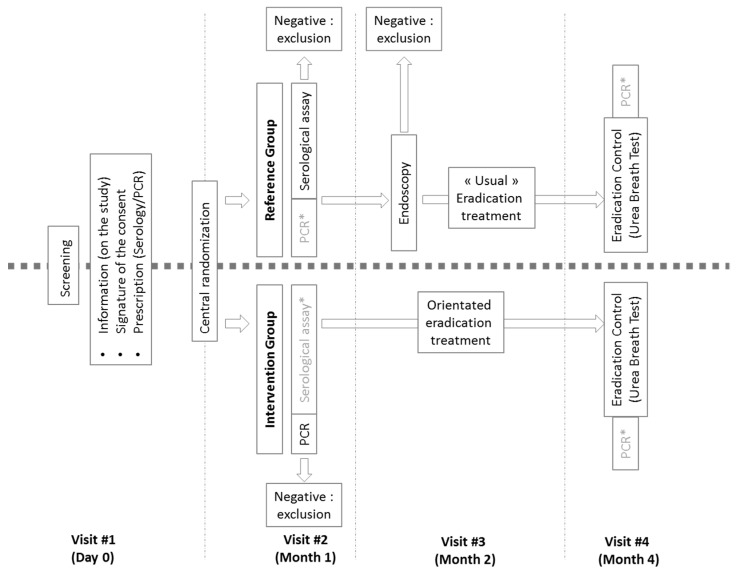
Trial design. *: Data collected for the study, the results of which will not be communicated to the patient and the physician before the end of the study.

This study will be a prospective, multicenter, randomized, controlled interventional study in two arms (Figure 1). Randomization will be carried out through a secure web-based randomization system and stratified by center (i.e., each investigator). The duration of the inclusion period will be 12 months. The participation period for each participant will be 4 months for a total study duration of 16 months (twelve months of inclusion plus four months of follow-up for the last included patient).

A computer-generated block-randomization sequence using permuted-block randomization of varying block sizes will be performed by the statistician and will be carried out via Ennov System software. The statistician will not be involved in either screening the patients or assessing outcomes. The randomization process will be accessible to all physicians working in the emergency department through user personal identification logins to access the website (https://chu-poitiers.hugo-online.fr/CSOnline/, accessed on 27 December 2021). It will become effective following confirmation of inclusion and exclusion criteria. Thereafter, patients will be assigned to one of the two study groups (in a 1:1 randomization scheme) according to the strategy (described thereafter).

**Intervention group.** Patients randomized to the Intervention group (new strategy arm) will receive a prescription for Hp serology with instructions for the laboratory to limit the communication of the results to the physician alone and a stool self-sampling kit (containing instructions, biodegradable basket to be placed on the toilet, e-Nat tube (Copan, Italia), and stamped envelope for mailing to the laboratory). After centrifugation, 400 μL of the eNAT tube supernatant will be extracted using the NucliSENS easyMAG system (bioMérieux, Marcy-l’Étoile, France), according to the manufacturer’s instructions, in 2 mL lysis buffer under the following conditions: the specific B 2.1 protocol with 140 μL of silica, after adding 1 μL of internal control (Amplidiag Easy Process Control I; Mobidiag, Hologic inc.), for a final volume of eluate of 70 μL. Nucleic acid will then be amplified using the Amplidiag *H. pylori* + ClariR test assay. This test, the only one that will be considered for this arm, consisting in a multiplex PCR, will obtain three different results (negative/absence of detection of *Helicobacter pylori* or detection of *Helicobacter pylori* or detection of *Helicobacter pylori* associated with mutation implicated in clarithromycin-resistance, i.e., A2142C, A2142G and A2143G). Originally developed for detection in gastric biopsy this kit may give appropriate results in stool samples [10,24]. According to the results of their biological assay, patients will be seen by their investigator within six weeks to either initiate or not initiate eradication therapy, i.e., a positive test will determine Hp infection and indication for treatment. The absence of detection of a clarithromycin-resistant strain will allow prescription of a guided treatment combining PPI, amoxicillin, and clarithromycin for 10 days according to the HAS recommendations. According to the same recommendations, the detection of a clarithromycin-resistant strain will allow prescription of a guided treatment combining PPI, amoxicillin, and metronidazole or quinolone for 10 days. At least 4 weeks after stopping antibiotics (and at least 2 weeks after stopping PPIs), an eradication control will be carried out using a respiratory test (urea breath test), concomitant to the collection of a stool sample for retrospective analysis by real-time PCR. A negative breath test will define the success of the treatment, while a positive test will define treatment failure. The latter will be managed in the usual manner by the GPs in charge of the patient. At the end of the follow-up, infected (positive for serology and/or PCR assays) and treated patients over 45 years of age will be referred to a gastroenterologist for gastric biopsies for histological analysis of the gastric mucosa for pre-neoplastic and cancerous lesions.

**Control group.** Patients randomized to the reference strategy control group (HAS strategy arm) will receive a prescription for Hp serology and a stool self-sampling kit (instructions, biodegradable basket to be placed on the toilet seat, E-Nat tube (Copan), swab, stamped envelope for mailing to the laboratory). Only the serology result will be considered for this arm (to avoid any deviation from the protocol, results of other tests will not be communicated before the end of the study). According to the results of their biological assay, patients will be seen by their investigator within six weeks. A positive serological test will lead to a gastroscopy with biopsies for histology/pathological and bacteriological analysis if available. Each person referred will be accompanied by the “request for gastroscopy in case of positive Hp serology” letter, according to the HAS model. These anatomopathological and/or bacteriological analyses will determine the presence of a Hp infection, if bacteria suggestive of Hp are observed in pathological examination and/or if the conventional and/or molecular biology are positive from gastric biopsies sent to bacteriology laboratories. An infection will determine the indication for treatment, either empirically, in the absence of an antibiogram or PCR result (bismuth or concomitant quadritherapy), or guided by the results of the AST or culture according to HAS recommendations (pertinence of care sheet). At least 4 weeks after stopping antibiotics (and at least 2 weeks after stopping PPIs), an eradication control will be carried out using a respiratory test (urea breath test), concomitant to collection of a stool sample for retrospective analysis by PCR. A negative breath test will define the success of the treatment, while a positive test will define treatment failure. The latter will be managed in the usual manner by the GPs in charge of the patient.

It should be noted that for ethical and regulatory considerations, it was decided, after the collection of results (at the end of the study for each patient), to give out the results of these analyses (urea breath test, serology, PCR before/after treatment) in order to allow the general practitioner to manage the patient in an appropriate manner.

### 2.2. Participant Eligibility and Consent

**Figure 2:**
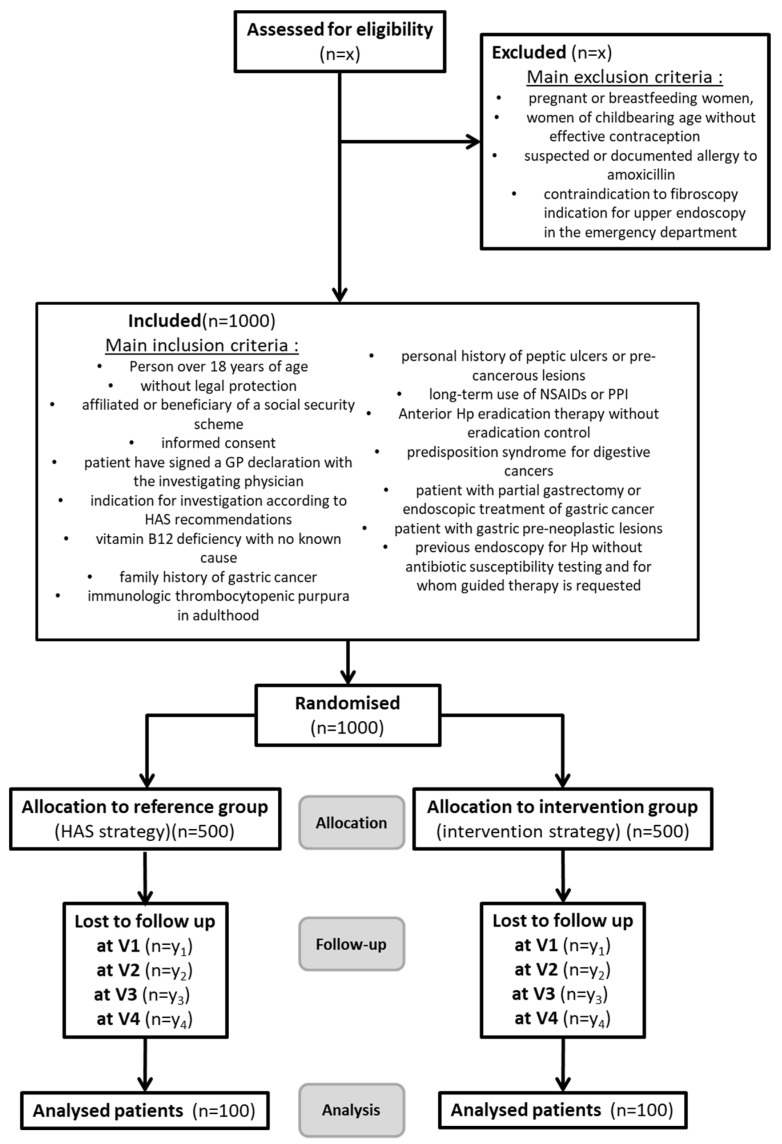
CONSORT Flow chart of the HepyPrim Study. X: data could not be predicted before the beginning of the screening. Y (= y1 + y2 + y3 + y4), corresponding to patients lost to follow-up, will be less than 50 patients per allocation group.

All consecutive patients will be considered candidates for inclusion in the study if they meet all inclusion criteria and none of the exclusion criteria (Figure 2.). Eligible patients will receive oral and written information and will be enrolled after giving written informed consent.

**Inclusion criteria.** Inclusion criteria will be: (i) over 18 years of age, (ii) affiliated to or beneficiary of a social security scheme, (iii) informed consent signed after clear and fair information about the study, (iv) patient having signed a GPs declaration with the investigating physician, (v) indication for investigation and treatment of Hp infection according to HAS recommendations (suffering from chronic dyspepsia, iron deficiency anemia with no known cause or resistant to iron supplementation, vitamin B12 deficiency with no known cause, family history of gastric cancer, immunologic thrombocytopenic purpura in adulthood, personal history of peptic ulcers or pre-cancerous lesions that have not been eradicated, long-term use of NSAIDs or PPI, patient who received Hp eradication therapy without eradication control, patient with a predisposition syndrome for digestive cancers (hereditary non-polyposis colorectal carcinoma cancer)), (vi) patient with partial gastrectomy or endoscopic treatment of gastric cancer, (vii) patient with gastric pre-neoplastic lesions (severe atrophy and/or intestinal metaplasia, dysplasia), and (viii) patient with previous endoscopy for Hp without antibiotic susceptibility testing (biopsy not referred to bacteriology) and for whom guided therapy is requested.

**Non-inclusion criteria.** Patients who will not be included are those who: (i) are legally protected; (ii) are pregnant or breastfeeding women or women of childbearing age without effective contraception; (iii) are suspected of or have a documented allergy to amoxicillin; (iv) have a contraindication to (or initial refusal of) esophageal endoscopy; and (v) have an indication for upper GI endoscopy in the emergency department according to the criteria of the European Panel on the Appropriateness of Gastrointestinal Endoscopy (i.e., upper GI bleeding, acute deglobulation without externalized GI bleeding, caustic ingestion, acute dysphagia or foreign body ingestion).

### 2.3. Study Outcomes

**Primary endpoint.** The primary endpoint will be an assessment of the cure rate through the results of a urea breath test performed 6 weeks after the end of treatment (proof of Hp eradication).

**Secondary endpoints.** The secondary endpoints will be assessment of: (i) the number of guided treatments (depending on the real-life application of the French authorities recommendations); (ii) the number of endoscopies (estimating the number of endoscopies avoided due to the optimized process); (iii) the number of biopsies at endoscopy; (iv) the number of patients who refused endoscopy after serology or positive PCR or did not follow-up after referral to the gastroenterologist; (v) the number of treatment side-effects and treatment dropouts; (vi) the discordance rate between real-time PCR tests in stool and serology in primary diagnosis; (vii) the discordance rate between real-time PCR tests in stool/breathing test in eradication control; and (viii) cure rate assessed by the result of a urea breath test performed 6 weeks after the end of treatment (proof of Hp eradication) depending on the antibiotic susceptibility testing identified at diagnostic.

### 2.4. Data Collection

Independent clinical research assistants will be available at each participating hospital to help in running the study and with data collection. Study documents will be deidentified and stored for 15 years, as per the protocol for non-clinical trial notification interventional studies. Data will be entered into the web-based eCRF (https://chu-poitiers.hugo-online.fr/CSOnline/, accessed on 27 December 2021) and electronically stored on double password-protected computers. Hard copies of data (clinical research files) will be stored in a secure locked office. All personnel involved in data analysis will be masked to study groups. Only the principal investigators and the statisticians will have access to the final data set.

The following data will be recorded:

**Baseline characteristics.** (i) Demographic data (age, gender, height, weight, and body mass index); (ii) comorbidities (active smoking, insulin-dependent or non-insulin-dependent diabetes, hypertension, hypercholesterolemia, chronic renal failure, COPD); (iii) history of antimicrobial treatment for eradication of *H. pylori*; (iv) key laboratory findings (except for Hp); (v) familial history of cancer; and (vi) symptomatology (including clinical examination).

**Biological data.** Results of the following biological assays: (i) Results of the stool PCR searching for *H. pylori* (before and after eradication treatment); (ii) serological status of the patient regarding *H. pylori*; (iii) histopathological results (for HAS groups); and (iv) results of the urea breath test.

### 2.5. Statistical Analysis

**Sample size calculation.** Assuming 20% prevalence in source population, and based on a one-sided hypothesis, 100 patients will be included in each strategy arm to demonstrate a 15% difference between each strategy with statistical risks at 5% (type I error, α) and 15% (type II error, β). We are planning to enroll 1000 patients to consider a maximum patient loss of 10%. Considering that each investigator involved in the HepyPrim study follows more than 1200 patients each year, the inclusion objective was set to 50 patients per investigator and the number of investigators was set to 20. These investigators have been selected in the newly constituted “physicians’ network for research in New Aquitaine”.

**Analysis.** The data will be analyzed blindly on an intention-to-treat basis, assuming a non-inferiority hypothesis. No interim analysis is planned. A per protocol analysis will be performed as a secondary sensitivity analysis. Quantitative variables will be described by their mean and standard deviation or median and interquartile range according to the normality of the distribution. Categorical variables will be described by their number and percentage. Continuous variables will be compared by a Student’s *t*-test or Mann–Whitney U-test according to the normality of the distribution. Categorical variables will be compared by a chi-square test or Fisher’s exact test. The tests will be analyzed using GraphPad Prism version 9.0 (GraphPad Software, San Diego, CA, USA). A significant difference will be retained for a *p*-value < 0.05. All tests will be two-tailed, stratified by center and for multiple comparisons if appropriate.

### 2.6. Ethical Considerations

The clinical trial will be carried out in line with the principles of the Declaration of Helsinki, the guideline for Good Clinical Practice of the International Conference on Harmonization, in accordance with the French law No. 2012–300 of 5 March 2012 on research involving the human person and with the Clinical Trials Directives 2001/20/EC and 2005/28/EC of the European Parliament. All included patients will have consented to the protocol after an appropriate information session that includes processes of the study and the constitution of the biobank. As with all interventional studies in France and according to French legislation, the final protocol will be validated by a randomly selected ethical committee (Comité de Protection des Personnes, CPP).

The results of the study will be given to the participating GPs, referring physicians and medical community no later than 2 years after completion of the trial through presentation at scientific conferences and publication in peer-reviewed journals. The main manuscript will mention the name of the sponsor (French Ministry of Health), and all trial sites will be acknowledged. All investigators having included or followed participants in the study will appear with their names under ‘the HepyPrim investigators’ in an appendix to the final manuscript. Authorship will be completed in accordance with the guidelines of the International Committee of Medical Journal (ICMJ). No professional writer will be used.

## 3. Discussion

This study may be a major study to demonstrate the benefits of optimizing therapeutic management of Hp, through GP education and management. The patients included in the study may benefit from a reliable diagnosis without an invasive examination, use of a second diagnostic test (limiting the risk of false negative) with consideration of a possible positive result by the doctor and the systematic performance of a respiratory test to control eradication (underused in France despite recommendations).

This study may make it possible to assess the interest of a non-invasive procedure for the diagnosis of *Helicobacter pylori* infections that can be carried out in primary care practice in France, coordinated by general practitioners. This study may make it possible to achieve better efficiency in recourse to gastroenterologists and endoscopy by limiting it, in the indication of endoscopies of the upper digestive tract, to complex cases, thereby optimizing interdisciplinary collaboration. The aim of this study is to promote the appropriation of “guided” treatments in the care of Hp infection by primary care in future coordinated multidisciplinary practice, while also promoting the prevention of high-prevalence and potentially serious pathologies of the upper digestive system.

The main challenge in curing Hp is associated with the management of antimicrobial resistance, particularly to clarithromycin, a key component of triple therapy and impacted by increasing resistance (up to two-thirds of bacterial strains isolated) [13,25,26,27]. This situation justifies the crucial need for biological tests capable of determining the antimicrobial susceptibility profile [21]. To this end, detection by molecular analysis of Hp DNA on stools is crucial to develop Hp-guided therapeutic strategies. Its non-invasive nature would allow for easy sample acquisition (with improved compliance), thus optimizing the time-to-cost importance of the disease. With the exception of recent large studies, most of the few studies have used small numbers of patients, tests adapted to stool samples but designed for biopsies (GenoType HelicoDR (Hain Lifescience, Nehren, Germany) or *H. pylori* ClariRes (Ingenetix, Vienna, Austria) [28,29,30,31,32,33,34,35]. On the contrary, two recent studies demonstrated the very high performance of the stool test for the detection of Hp infection and clarithromycin resistance, in a process that takes little time [10,36].

Moreover, this is the first study to enable the structuring of a research network in primary care in Poitou-Charentes. This project is the first of this network, which will be made up of 20 co-investigators, all of whom are GPs. Others were identified during the recruitment phase carried out as part of a thesis on the practice of general medicine by two interns in general medicine. This project is therefore founding and structuring for a primary care research network.

This study may allow evaluation of the performance of PCR in stools in comparison with the combination of serology/pathology/bacteriology for the initial diagnosis, evaluation of the performance of PCR in stools in comparison to the respiratory test for the control of eradication. Moreover, it may lead to the constitution of a biobank of stools of infected patients, in view of further study of the development of in vitro diagnosis.
